# On the Response of Halophilic Archaea to Space Conditions

**DOI:** 10.3390/life4010066

**Published:** 2014-02-21

**Authors:** Stefan Leuko, Petra Rettberg, Ashleigh L. Pontifex, Brendan P. Burns

**Affiliations:** 1 Deutsches Zentrum für Luft- und Raumfahrt, Institut für Luft- und Raumfahrtmedizin, Abteilung Strahlenbiologie, Arbeitsgruppe Astrobiologie, Linder Höhe, Köln 51147, Germany; E-Mails: stefan.Leuko@dlr.de (S.L.); petra.rettberg@dlr.de (P.R.); 2 School of Biotechnology and Biomolecular Sciences, University of New South Wales, Sydney NSW 2052, Australia; E-Mail: a.pontifex@student.unsw.edu.au; 3 Australian Centre for Astrobiology, University of New South Wales, Sydney NSW 2052, Australia

**Keywords:** halophilic archaea, space conditions, UV

## Abstract

Microorganisms are ubiquitous and can be found in almost every habitat and ecological niche on Earth. They thrive and survive in a broad spectrum of environments and adapt to rapidly changing external conditions. It is of great interest to investigate how microbes adapt to different extreme environments and with modern human space travel, we added a new extreme environment: outer space. Within the last 50 years, technology has provided tools for transporting microbial life beyond Earth’s protective shield in order to study *in situ* responses to selected conditions of space. This review will focus on halophilic archaea, as, due to their ability to survive in extremes, they are often considered a model group of organisms to study responses to the harsh conditions associated with space. We discuss ground-based simulations, as well as space experiments, utilizing archaea, examining responses and/or resistance to the effects of microgravity and UV in particular. Several halophilic archaea (e.g., *Halorubrum chaoviator*) have been exposed to simulated and actual space conditions and their survival has been determined as well as the protective effects of halite shown. Finally, the intriguing potential of archaea to survive on other planets or embedded in a meteorite is postulated.

## 1. Introduction

Earth is currently the only known planet harboring life, while the rest of the solar system appears to be both hostile and uninhabitable. Conversely it is important to note that it was long believed that extreme environments on Earth were “dead zones”, in which no form of life could be found. However, in the last four decades, scientists have discovered a remarkable diversity of organisms from all three kingdoms of life in these “extreme” environments. New organisms are continually discovered and broaden our knowledge of extremophiles and their respective environments. However, our quest to discover new extreme environments and conditions is no longer limited to our planet. Since 1953, when the term “Astrobiology” was coined in the Soviet Union [1], scientists have aimed to address different queries in terms of astrobiological research that relate to microbiology: (i) Are microbes able to survive space or simulated extraterrestrial environments? (ii) Could humans utilize microbes during manned missions to other planets? (iii) Do microbes pose a threat to humans due to biological contaminations? and (iv) The risk of forward contamination. The ability of microbes to survive space travel is of particular concern for Mars surface missions, as a number of terrestrial organisms can survive in a simulated Mars environment when protected from solar UV radiation [2,3,4] and subsequently compromise further studies for detection of past and present life [5]. The survival abilities of microbes are also of interest for the controversial theory of Lithopanspermia, the postulated transfer of microbes in rocks from one planet to another.

In this review, we will discuss what insight and information the archaeal family *Halobacteriaceae* can provide on the above formulated questions. Several ground based experiments, as well as space experiments, have already been conducted and will be discussed in detail. Furthermore, the ability of halophilic archaea to survive on other planets or embedded in a meteorite is postulated.

## 2. Characteristics and Environments of Halophilic Archaea

The family *Halobacteriaceae* was established [6], to accommodate the genera *Halobacterium* and *Halococcus* [7]. As of November 2011, the family consists of 129 species whose names have been validly published, classified in 36 genera [8]. The term “halophilic” is generally restricted to those that have a specific requirement for salt. Organisms termed halophilic will not grow in the absence of high salt concentrations, usually greater than 1.0–1.5 M NaCl [9]. Halophilic archaea belong to the phylum Euryarchaeota, are chemoorganotrophic and thrive in a wide array of environments such as the Dead Sea [10,11], solar salterns [12,13,14], and have been isolated frequently from subterranean salt cores [15,16,17]. Halophilic archaea have also been isolated from permanently cold evaporation ponds found in the dry regions of Antarctica e.g., Deep Lake [18,19]. More unusual environments for halophilic archaea, due to relatively low sodium chloride content, are modern stromatolites located in Shark Bay [20,21], Zodletone Spring [22] and even the nostrils of the seabird *Calonectris diomedea* [23].

Haloarchaea are a group of organisms with many unusual features. Some of these characteristics include an ability to grow at saturated salinity, possess a striking pigmentation in red, orange or purple, have obligate salt-dependent enzymes and possess a unique proton pump, bacteriorhodopsin, which is driven just by sunlight [24]. The cell morphology ranges from rod, cocci and irregular pleomorphic forms [24] to the very unusual structure of “*Haloquadratum walsbii*”, which exhibits an almost perfect quadratic cell form [25]. Lipids of halophilic archaea, are distinctly characterized by ether linkages and isoprenoid chains, mainly phytanyl and bis-phytanyl, in contrast to the ester linkages and straight fatty acyl chains of non-archaea [26]. Most notably, halophiles contain a major ubiquitous phospholipid, archaetidylglycerol methylphosphate (PGP-Me), which accounts for 50%–80% of the polar lipids and it is a major contributor of membrane stability in extreme environments [26,27]. Perhaps the most striking feature of this group is the potential longevity of halophilic microorganisms in salt sediments for millions of years.

## 3. Stress Resistance of Halophilic Archaea on Earth

Halophilic archaea have been investigated in great detail in regards to their response to different stress situations. The best-studied representative to date is the laboratory strain *Halobacterium salinarum* NRC-1. Studies have investigated how this strain reacts to desiccation [28], shifts in osmotic pressure [29,30], heat [31], oxidative stress [32], ionizing radiation [28,33,34], oxygen limitation [32], and a broad range of different UV radiation regimes [35,36]. Other representatives of the halophilic archaea, such as *Halococcus hamelinensis*, have been investigated based on their osmoadaptive strategies [37] and their resistance to UV-C radiation and the subsequent repair [38]. It has also been shown that the UV resistance of halophilic archaea increases dramatically when embedded in halite [39], and that intracellular salts provide protection against ionizing radiation in *Halobacterium salinarum* NRC-1 [40]. All the previous experiments and results clearly suggest that halophilic archaea possess sophisticated mechanisms to survive particular stress conditions and are therefore ideal candidates for space related studies.

## 4. Simulated Space Conditions

Although tremendous advances have been made in the quest to explore outer space, the ability to send biological samples to outer space are extremely difficult to realize and pose a number of challenges. For a cost-effective and feasible way to gain insights into the effect of space conditions on organisms, carefully designed ground-based simulation experiments have been conducted*.* Koike and colleagues studied the resistance of *Halobacterium halobium* to a simulated Martian atmosphere only to find that this organism is not able to survive such conditions unprotected [41]. One possible explanation for this result is that they exposed the strain to UV and proton radiation that correspond to about 200 years on Mars [41]. Another space relevant experiment was conducted by the group of Stan-Lotter [42], where *Hbt.* *salinarium* NRC-1 and *Halococcus dombrowskii* were exposed for 6 h to simulated Martian conditions. Results suggested that *Halococcus dombrowskii* is somewhat more resistant to exposure to extreme environments—by a factor of about 10 under the conditions tested—than *Hbt. salinarium* NRC-1, yet it was possible to recover both strains following exposure to a simulated Martian atmosphere. It needs to be taken into consideration that for those exposure experiments, samples were exposed to normal daylight, and not UV light.

Other ground-based experiments have employed both *Halococcus dombrowskii* and *Haloferax mediterranei*, in which they were grown in simulated microgravity (SMG) for several days and the consequent effects were observed [43]. Both strains showed an increased resistance against antibiotics and there were several differences in the proteome following growth in SMG. Another interesting occurrence whilst being grown in SMG was that cell aggregation did not occur in comparison to normal growth conditions. Cell aggregation appears to be strongly affected when environmental changes occur, for example the Antarctic archaeon *Halorubrum lacusprofundi* exhibits a distinct change in morphology at low temperatures, clumping in aggregates embedded in a network of fibrils [44].

## 5. Halophilic Archaea in Space

### 5.1. BIOPAN Mission

The first halophilic archaeon to be exposed to outer space was *Halorubrum chaoviator* sp. strain Halo-G* onboard the BIOPAN facility (used for short-term exposure) launched in 1994 [45,46]. The BIOPAN capsule was a small retrievable capsule developed by the European Space Agency (ESA) for the exposure of biological and chemical samples in low Earth orbit. For this experiment, *Hrr. chaoviator* was prepared unprotected or embedded in clay, meteorite powder, simulated Martian soil, or salt crystals, dried onto quartz discs and sent into outer space by a Russian spacecraft of the Foton class [2,45]. Once in space, the motor-driven hinged lid opens 180° in orbit to expose the samples to space vacuum, UV and cosmic radiation. For re-entry, the closed facility is protected with an ablative heat shield. The capsule then circled the planet in a low Earth orbit for two weeks, with follow up investigations displaying how *Hrr. chaoviator*, protected by the previously mentioned materials, survived this outer space exposure [45].

### 5.2. EXPOSE-E and EXPOSE-R

The EXPOSE facility was designed by ESA for medium and long-term exposure experiments on the outside of the ISS (International Space Station) (for a detailed overview of the facility refer to [47]). Comparable to the BIOPAN facility, experiments are dried onto different carrier material (e.g., quartz discs) and located in tiny cells that are pressurized or vented, protected with windows and filters of various geometrics and materials [2,48]. The EXPOSE assembly includes the facility and its supporting structure, interfacing with the EUTEF—CEPA (Columbus External Platform Adapter) for EXPOSE-E or with the external platform of the Russian segment of the ISS for EXPOSE-R [2].

The EXPOSE-E mission (EXPOSE-E trays were attached to the EuTEF) was launched on 7 February 2008 onboard US NASA Space Shuttle Atlantis, STS-122. In addition to chemical experiments, the EXPOSE-E trays carried several different organisms, one of which was *Halococcus dombrowskii* as part of the ADAPT (Molecular adaptation strategies of microorganisms to different space and planetary UV climate conditions) experiment. *Hcc. dombrowskii*, an isolate from Permian salt sediments in Austria [16], was exposed with the other experiments for 559 days to the space environment. EXPOSE-E was only used for one mission, as it had no removable experimental trays and was therefore part of the EUTEF and was dismounted with the samples [2] and returned to Earth. At this point, there is no publication on the results of this experiment so it is unclear if *Hcc. dombrowskii* was able to survive this particular experiment.

EXPOSE-R was successfully launched on 26 November 2008, with the Russian Progress 31-P. This mission accommodated eight experiments, one of which was the ROSE2/OSMO experiment in order to understand the response of *Synechococcus* and *Haloarcula-G* to the space environment. EXPOSE-R possesses removable trays and the facility on the Russian segment is still outside the ISS and will be used for further missions e.g., EXPOSE-R2. Similar to the EXPOSE-E mission, there are no current publications on the results, highlighting the pit-falls associated with actual space experiments. Although experimental verification of halophilic archaea surviving outer space conditions is limited, another equally interesting question can be raised in relation to halophilic archaea and their potential ability to survive on another planet.

### 5.3. Exoplanets

When looking for possible habitable exoplanets in respect to halophilic archaea, such a planet would have to have two major environmental factors present: water and salt. It is well-known that hypersaline brines can extend the range and stability of liquid water on Earth such as Deep Lake Antarctica, where halophilic archaea have been isolated [18,19]. Similar brines may exist on exoplanets that have extremely hostile surface conditions e.g., the Jupiter moon Europa [49] or on Mars.

In the Solar System, Mars and Earth are neighbors and are therefore most likely to share certain early geological processes [50,51]. Many previous studies draw a picture of early Mars as being a warmer and wetter planet with a substantial amount of water earlier in its history [52,53,54], indicating the possibility of hypersaline brines on early Mars [55]. More recently, evidence for water vapor in excess of saturation in the atmosphere has been shown [56], and it has also been highlighted that Mars is a salt rich environment [57]. It is therefore intriguing to postulate that although Mars today is an inhospitable planet for life, halophilic archaea may have been enclosed in one of those brines and lying dormant over geological time there ever since.

The Jupiter moon Europa is also considered a potential host for extraterrestrial life within our Solar System. Although inhospitable on the surface, it is believed that it may harbor a global salty liquid water ocean with two to three times the volume of all liquid water on Earth below the surface [58,59]. This ocean lies between a relatively thin ice crust and a silicate mantle and tidal heating has been suggested as the power source maintaining this ocean [60,61]. This liquid water, in combination with its silicate seafloor and radioactively produced surface oxidants, may provide for a chemical rich ocean that could be considered habitable by terrestrial standards [58,62]. Taking all the properties of the subterranean ocean, a predicted abundance of NaCl, KCl and MgCl_2_ [58] into account, Europa would make an interesting candidate to look for halophilic life.

Enceladus, a small icy moon of Saturn, also has an ocean underneath an ice cover with images from the Cassini mission revealing about a dozen jets of fine icy particles that emerge from the south polar terrain and feed a giant plume extending thousands of kilometers into space [63,64]. The composition of this plume and other assumptions led some scientist to believe that an early ocean on Enceladus was an alkaline Na^+^-Cl^−^-HCO_3_^−^ solution [65]. A modern ocean, if an aqueous phase still exists on Enceladus, could consist of a eutectic Na-Cl-HCO_3_ brine that may facilitate tidal heating, which is required to sustain life.

Although life on these planets and moons would be possible under certain circumstances, no trace of life has yet been found on either of them. However, further explorations and refined techniques for the detection of life may alter that.

### 5.4. Interstellar Travel Onboard Meteorites

Once entrapped in halite, it has been postulated that halophilic archaea may be able to travel through outer space onboard meteorites [66,67,68]. Although halite has been discovered in meteorites, it is still unclear if entrapped halophilic archaea would be able to survive the planetary ejection and the subsequent atmospheric entry. Further studies are needed to test the feasibility of halophilic archaea trapped in halite and their viability when exposed to forces similar to ejection and re-entry.

## 6. Conclusions and Future Space Missions

Ground based experiments complement the exo/astrobiological experiments conducted in the lower Orbit or in outer space. One example is the ESA accepted space simulation for investigating organics, evolution and exobiology (SSIOUX) experiment, where an international consortium of scientists will expose organic compounds and a wide range of microorganisms to simulated space parameters in pursuit of exobiological questions on their resistance in the space environment and the origin and distribution of life [69]. Further space flight experiments are currently in preparation, such as the BOSS (Biofilms in Space) experiment. This proposal tests the hypothesis that *Halococcus morrhuae* will survive the space environment better when co-exposed with the biofilm forming bacterium *Halomonas muralis*. Both have been found on the same mural painting in a castle in Austria [70,71]. Samples will be dried onto quartz discs and exposed during the EXPOSE-R2 mission that is scheduled to launch in 2014 and will stay outside the ISS for 1.5 years.

With human presence in space growing, it is of key interest to fully understand how different microorganisms react to the hostile environment of outer space. With all the proposed ground and flight experiments currently in preparation, it is an exciting time to be in this field, with the likelihood of further novel and ground-breaking discoveries.
